# The Impact of Zinc Supplementation on Hyperglycemia and Complications of Type 2 Diabetes Mellitus

**DOI:** 10.7759/cureus.73473

**Published:** 2024-11-11

**Authors:** Naina Mehta, Mohamad Akram, Yogesh P Singh

**Affiliations:** 1 Internal Medicine, Swami Rama Himalayan University, Dehradun, IND; 2 Clinical Immunology, All India Institute of Medical Sciences, Bilaspur, IND

**Keywords:** diabetic microvascular complications, diabetic retinopathies, “insulin resistance”, types 2 diabetes mellitus (t2dm), zinc supplementation

## Abstract

Introduction: Type 2 diabetes mellitus (Type 2 DM) constitutes a major public health problem. Zinc (Zn), a critical micronutrient in the human body, serves as a potent antioxidant, and is closely linked to the development and progression of Type 2 DM. However, limited evidence explored the speculated putative mechanism of Zn repletion improving insulin sensitivity and severity in patients with Type 2 DM.

Methods: Adult participants (aged more than 18 years) diagnosed with Type 2 DM (with or without microvascular complications) were recruited. A case recording form divided into Section A, socio-demographics, and Section B, clinical parameters, a brief clinical history and lab investigations, were logged for each patient.

Results: Majority of patients (n=44, 47.8%) were over 60 years old, with a nearly equal distribution of sex (n=47, 51.1% male and n=45, 48.9% female). Sensory motor (n=50, 54.3%) and visual disturbances (n=48, 52.2%) were the most commonly reported symptoms. Oral hypoglycaemic agents (OHA) were the most common drug treatment (n=56, 60.9%, p<0.001). A considerable number of patients had systemic hypertension (n=55, 59.8%) and neuropathy was the most prevalent complication (n=68, 73.9%), followed by retinopathy (n=53, 57.6%). Zn deficiency was prevalent in n=50, 54.3% of patients and it showed significant associations with poor glycaemic control, diabetic neuropathy (<0.001), retinopathy (<0.001), and nephropathy (<0.001).

Conclusion: We highlighted the high prevalence of Zn deficiency among Type 2 DM patients and established significant correlations between low Zn levels (<65 μg/dL) and poor glycemic control, as well as higher incidences of neuropathy, retinopathy, and nephropathy. We advocate that Zn status should be considered in the management and treatment strategies for Type 2 DM patients to potentially mitigate complications and improve outcomes.

## Introduction

Type 2 diabetes mellitus (Type 2 DM), a chronic metabolic disease characterized by elevated levels of blood glucose/sugar alongside impaired carbohydrate, lipid, and protein (due to insufficient insulin secretion), constitutes a major public health problem [1]. Worldwide, it has a prevalence of approximately 422 million (mostly in low- and middle-income countries) and is responsible for 1.5 million deaths yearly [2]. Currently, 347 million adults worldwide have Type 2 DM; with its prevalence continuing to rise, it is expected to be the seventh leading cause of death by 2030 presumably due to population aging, growth, urbanization, low physical activity and the high prevalence of obesity [2]. In South-East Asia, Type 2 DM affected nearly 8.8% of adults in 2019, totalling 87.6 million people; notably, a significant number remain undiagnosed [3]. Specifically, India faces a burgeoning Type 2 DM epidemic harbouring 77 million diabetics aged 20-70 years primarily driven by rapid urbanization, sedentary lifestyles, unhealthy diet, and tobacco use [4,5]. Indeed, these staggering numbers underscore the urgent need for comprehensive healthcare strategies to address Type 2 DM, its potential complications and contributing factors.

Clinically, the early symptoms of untreated Type 2 DM are related to elevated blood sugar levels and extension of it into the urine. High amounts of glucose (>0.25mg/ml) in the urine can cause polyuria leading to dehydration which causes increased thirst and water consumption i.e. polydipsia [6]. At times, Type 2 DM patients may complain of fatigue, nausea and vomiting [6]. Other associated symptoms include weight loss and blurred vision. The long-term macrovascular and microvascular complications of Type 2 DM range from cerebrovascular accidents, peripheral arterial disease, and coronary heart disease to peripheral neuropathy, diabetic retinopathy, and nephropathy [7]. Chronically, diabetic retinopathy potentially results in loss of vision, nephropathy leading to renal failure, and peripheral neuropathy poses risk of foot ulcers, amputation and autoimmune neuropathy resulting in gastrointestinal, genitourinary and cardiovascular symptoms [2,6]. In Type 2 DM patients, one of the postulated pathophysiological mechanistic theories is chronic hyperglycaemia increases oxidative stress by the production of free radicals (oxidants), plus the reduction of the antioxidant defence system. This leads to oxidative cellular injury resulting in cellular dysfunctions [2,6].

Zinc (Zn), as a critical micronutrient in the human body, serves as a potent antioxidant that plays a significant role in mitigating oxidative stress. Its antioxidative action is crucial for maintaining cellular health and preventing the damage that oxidative stress can cause to cells and tissues. The lack of adequate Zn levels (<70 mcg/dL) is closely linked to the development and progression of chronic diseases, including Type 2 DM. In individuals with Type 2 DM, the concentration of Zn in the body can be adversely affected through mechanisms such as increased excretion and diminished absorption from the intestines, as well as enhanced renal excretion, which further exacerbates the body's Zn deficiency [7]. Its mechanism of action is multifaceted, particularly in its relationship with insulin, the hormone central to glucose metabolism. Potentially, Zn acts as a cofactor necessary for the synthesis, storage, and possibly the secretion of insulin from the pancreas. In the context of Type 2 DM, where insulin resistance is the predominant issue, Zn deficiency can potentially aggravate the condition, highlighting the mineral's importance in insulin function and glucose homeostasis [8]. Extensive molecular and cellular research underscores Zn's pivotal role not only in insulin synthesis/function but also in the broader metabolic processes affected by Type 2 DM. Observational studies have drawn correlations between lowered serum Zn levels and the presence of established Type 2 DM, suggesting a critical link between Zn status and diabetes management. Although rodent models of Type 2 DM have shown the beneficial impacts of Zn supplementation in moderating the disease's progression, the efficacy and safety of such supplementation in humans require further exploration through well-designed clinical trials [6,9,10]. Zn depletion has several potential clinical implications. It is speculated that Zn repletion could improve insulin sensitivity in patients with Type 2 DM and reduce the severity of certain complications of this disease.

As such, to understand the underlying patho-biochemical inter-relationships of the complications of Type 2 DM with Zn levels in more detail, this study is being undertaken with two distinct aims: (1) to assess the relationship between level of Zn and hyperglycaemia in Type 2 DM, and (2) To assess the relationship between level of Zn and microvascular complications of Type 2 DM such as diabetic neuropathy, diabetic retinopathy and diabetic nephropathy. Our hypothesis is that Zn supplementation is helpful in the prevention of Type 2 DM and its related complications, as stated above. 

## Materials and methods

Participants

Ninety-two (92) adult participants aged more than 18 years visiting the General Medicine out-patient department (OPD) and in-patient department (IPD) of the Himalayan Institute of Medical Sciences Hospital, who have been diagnosed with Type 2 DM with or without microvascular complications like diabetic retinopathy, diabetic neuropathy and diabetic nephropathy were recruited. Criteria for the diagnosis of Type 2 DM include one of the following: (1) fasting plasma glucose (FPG) ≥126 mg/dL (7.0 mmol/L) (Fasting is defined as no caloric intake for at least eight hours) or two-hour plasma glucose (PG) ≥200 mg/dL (11.1 mmol/L) during oral glucose tolerance test (OGTT) (This test was performed as per the WHO guidelines, using a glucose load containing the equivalent of 75 grams of anhydrous glucose dissolved in water), (2) Glycated hemoglobin (HbA1C) ≥6.5% (48 mmol/mol), (3) in patients with classic symptoms of hyperglycemia or hyperglycemic crisis, a random plasma glucose ≥200 mg/dL (11.1 mmol/L). Participants that include pregnant females, pre-diabetics or with Type 1 DM, those consuming multivitamins and/or health supplements such as anabolic steroids and/or ayurvedic/desi medicines, diagnosed with various enteropathies (e.g., acrodermatitis enteropathica) or malabsorptive disorders (e.g., celiac disease), malignancies, or on medications such as anti-tubercular treatment (ATT), oral contraceptive pills, valproate, penicillamine were excluded from the study. The washout period for participants taking supplements was six months per the European Food Safety Authority (EFSA) guidelines. Specifically, patients suspected with Type 1 DM were tested for C-peptide levels; if found below 5 µU/mL (0.6 ng/mL) they were excluded (Type 1 diabetes). 

Ethical approval

Institutional ethical approval from Himalayan Institute of Medical Sciences, Swami Rama Himalayan University, Jolly Grant, Dehradun, India, which conformed to the standards of the journal was granted prior to recruitment and testing and the study was conducted in accordance with the Declaration of Helsinki (REC Approval Code: SRHU/HIMS/RC/2024/238).

Experimental protocol

A case recording form divided into the following sections was used to collect the data: Section A: Socio-demographic details: Age of the patient, sex, marital status, pregnancy status of female subjects, familial status (joint family/unitary), educational status; Section B: Clinical parameters - a brief clinical history and lab investigations: comorbidities: hypertension, coronary artery disease, lab investigations: HBA1C levels using Boronated Affinity Lab Method, fasting blood sugar level, postprandial blood sugar level, serum Zn levels using inductively coupled plasma mass spectrometry (ICPMS) (Normal Reference Range: 65 - 256 microgram/dl), serum creatinine, serum K+, serum Na+, urine albumin, 24-hr urine protein, urine routine/microscopy, complete hemogram/erythrocyte sedimentation rate (ESR)/C-reactive protein (CRP). All blood samples were collected in the morning fasted and were processed on the same day. Specifically for zinc, the ICPMS instrument used was an Agilent 7900 ICP-MS (Agilent Technologies, Tokyo, Japan) connected to an SPS 4 Autosampler (Agilent Technologies), and data was recorded using the Agilent MassHunter Data software. To ensure apt quality control, R2 value for zinc was 0.9865 or 98.65%, and the calibration or validation range was 2.5 to 1,000 μg/L. The socio-demographic data and clinical details of the patients were recorded in the study pro forma. Fundus examination, bilateral lower limb nerve conduction velocity (NCV) study, and urinary albumin/24 hours. Urine protein were checked in all patients to look for diabetic retinopathy, neuropathy and nephropathy respectively. Details of investigations including a complete blood count, fasting blood glucose, postprandial blood glucose, HBA1C level, ESR, CRP, NCV of bilateral lower limb, serum creatinine, serum sodium, serum potassium, urinary albumin and urine routine microscopy were recorded in the study pro forma.

Sample size calculation

The following sample size formula was used for calculating the adequate sample size: n = (Z1-α/2)2P(1−P)/d2, where n is the sample size, Z is the standard normal deviation at 2-sided 95% confidence level = 1.96, P is expected prevalence. The minimum detectable diﬀerence based on previous work of prevalence (P) of Zn deficiency in T2DM - 40% or 0.40. The precision (d) has been taken as 10% or 0.10. N = 1.96 x 1.96 x 0.40 (1-0.40) / (0.10*0.10) = 0.921/0.01 = 92, the approximate number of participants required is 92. Since it's based on the prevalence (P) of Zn deficiency in Type 2 DM, it supports our results/conclusion, at least based on our sampling frame population.

Statistical analysis

Data was collected and entered Office Excel version 2010 (Microsoft, Redmond, WA, USA) and tabulated. SPSS software version 25 (IBM Corp., Armonk, NY, USA) was used to analyze the data that has been tabulated. A Shapiro-Wilk test was performed to assess the distribution of normality. The end results were described in the form of frequency charts, percentages, and Mean ± SD. Differences in participants’ demographics were analysed using independent-sample t-tests. An association between the Zn levels, Type 2 DM, and complications of Type 2 DM were analysed using Chi (χ) square test and Fisher exact test. Specifically, the Chi (χ) square test was used to compare differences in categorical variables, and ANOVA was used to compare mean values of continuous variables between groups. A 'p' value of <0.05 was considered statistically significant.

## Results

Demographic and general characteristics

In Table 1, the majority of the patients (SD: 4.3 years, n=44, 47.8%) were over 60 years old, followed by 46-60 years (SD: 5.8, n=40, 43.5%), and 31-45 years (SD: 3.4 years, n=8, 8.7%). There was a nearly equal distribution of gender among patients with n=47, 51.1% male and n=45, 48.9% female. Sensory motor disturbance (n=50, 54.3%) and visual disturbances (n=48, 52.2%) were the most commonly reported symptoms. Polyphagia was the least reported symptom (n=4, 4.3%). A significant portion of patients had a history of smoking (n=31, 35.9%) and alcohol intake (n=38, 41.3%). Most participants (n=82, 90%) belong to middle socio-economic status with BMI of 28.42 kg/m2 (SD ± 5.32).

**Table 1:** Demographic and general characteristics of patients with Type 2 diabetes mellitus

Demographic Characteristics	Frequency	Percentage (%)
Age (years ± SD)		
18-30 (0)	8	8.7
31-45 (3.4)	40	43.5
46-60 5.8)	44	47.8
>60 (4.3)	92	100
Sex		
Male	47	51.1
Female	45	48.9
Symptoms		
Polyuria	14	15.2
Polyphagia	4	4.3
Polydipsia	8	8.7
Oliguria	22	23.9
Visual disturbances	48	52.2
Sensory motor disturbance	50	54.3
History of smoking		
Yes	33	35.9
No	59	64.1
History of alcohol Intake		
Yes	38	41.3
No	54	58.7
History of systemic hypertension		
Yes	55	59.8
No	37	40.2
Socioeconomic Status		
Low	5	4.5
Middle	82	91
High	5	4.0

Clinical and biochemical profile of patients of type 2 DM

In Table 2, nearly half of the patients (n=46, 50%) had diabetes for five to 10 years, and 13 (14.1%) were newly diagnosed. Oral hypoglycaemic agents (OHA) were the most common treatment (n=56, 60.9%). Neuropathy was the most prevalent complication (n=68, 73.9%), followed by retinopathy (n=53, 57.6%).

**Table 2:** Clinical and biochemical profile of pateints of type 2 diabetes mellitus FBS: fasting blood glucose, PPGS: post prandial blood glucose, OHA: Oral hypoglycaemic agents, HbA1C: glycated hemoglobin

Clinical and biochemical characteristics	Frequency	Percentage (%)
Duration (Years)		
Newly Diagnosed	13	14.1
<5	15	16.3
5 – 10	46	50.0
>10	18	19.6
Blood glucose lab measurements		
FBS (mg/dl)	159.7 ± 52.7	90 – 332
PPBS (mg/dl)	242.9 ± 96.3	118 – 571
HbA1C (%)	9.2 ± 2.5	5.7 – 14.9
Complications		
Neuropathy	68	73.9
Retinopathy	53	57.6
Microalbuminuria	49	53.2
Abnormal Renal Function	37	40.2
Dyslipidemia	42	45.7
History of Treatment		
No Treatment	16	17.4
OHA	56	60.9
Insulin	10	10.9
OHA + Insulin	10	10.9

Relationship between level of zinc and glycaemic control in type 2 DM and its complications

In Table 3, most patients (n=44, 88%) with Zn level <65 μg/dL had uncontrolled diabetes (HbA1c > 7%). Zn deficiency (<65 μg/dL) showed significant associations with poor glycaemic control (p<0.001), diabetic neuropathy (p<0.001), retinopathy (p<0.001), and nephropathy (p<0.001). The majority of patients with neuropathy had axonal neuropathy (n=36, 85.7% and n=21, 80.8%). Patients with Zn deficiency (<65 μg/dL) exhibited higher rates of neuropathy, retinopathy and nephropathy, indicating a potential role of Zn in mitigating diabetic complications.

**Table 3:** Relationship between level of zinc and glycaemic control in type 2 diabetes mellitus and its complications – diabetic neuropathy, diabetic retinopathy, and diabetic nephropathy HbA1C: glycated hemoglobin

Zn level (μg/dL)	HbA1c Level (%)		P value
	≤ 7%; n (%)	>7%; n (%)	
<65	6 (12)	44 (88)	<0.001
≥65	19 (45.2)	23 (54.8)	
	Diabetic Neuropathy		
	Yes	No	
<65	42 (84)	8 (16)	<0.001
≥65	26 (61.9)	16 (38.1)	
	Type of Neuropathy		
	Axonal	Demyelinating	
<65	36 (85.7)	6 (14.3)	>0.05
≥65	21 (80.8)	5 (19.2)	
	Diabetic Retinopathy		
	Yes	No	
<65	36 (72)	14 (28)	<0.001
≥65	17 (40.5)	25 (59.5)	
	Diabetic Nephropathy		
	Yes	No	
<65	33 (66)	17 (34)	<0.001
≥65	16 (38.1)	26 (61.9)	

## Discussion

This study aims to assess the relationship between level of Zn and glycaemic control in Type 2 DM and to assess the relationship between level of Zn and microvascular complications of Type 2 DM such as diabetic neuropathy, diabetic retinopathy and diabetic nephropathy. We found a significant association between Zn levels (<65 μg/dL) and HbA1c levels (≥7%) in Type 2 DM patients (p <0.001). Furthermore, a significant association was observed between Zn levels and diabetic neuropathy, specifically, axonal neuropathy (p <0.001), diabetic retinopathy (p <0.001), and diabetic nephropathy (p <0.001) as compared to the patients with no Zn deficiency.

We found a significant association between Zn levels and HbA1c levels in Type 2 DM patients (p <0.001). Corroborative evidence (p<0.001) from prior research by Farooq et al. [11] aligns with the current study, further reinforcing the reliability of these findings. The prevalence of more than half the participants (n=50, 54.3%) falling below the recommended Zn threshold (<65 μg/dL) brings to the forefront the potential health implications associated with Zn deficiency. Indeed, our study cohort underwent a thorough dissection based on the duration of their Type 2 DM, with almost half (n=46, 50%) falling within the five to 10 years duration category (Table 1). This is in stark contrast as reported by Pan et al. [12], highlighting the unique demographic distinction inherent to divergent Type 2 DM cohorts under scrutiny. The outcomes of the present study pertained to the individuals (n=55, 59.8%) grappling with systemic hypertension closely aligned with those of a study conducted by Acharya et al. [13]. This correlation between the findings of the two studies lends credibility to the observed prevalence of systemic hypertension in the current investigation. Also, only one-third of patients (n=25, 27.2%) had controlled Type 2 DM signified by HbA1c level (≤ 7%) while the majority (67, 72.8%) had uncontrolled diabetes. Results of the present study bear semblance with the investigation conducted by Chadha et al. [14].

Analysis of our data related to Type 2 DM complications revealed that almost three-fourths of patients with Type 2 DM had diabetic neuropathy (n=68, 73.9%) that emerged as the most prevailing complication, followed by a two-thirds prevalence of diabetic retinopathy (n=53, 57.6%), and nearly half of the patients had diabetic (n=49, 53.2%). Upon further dissection, out of 68 patients with neuropathy, 57 patients (83.8%) had axonal neuropathy (Table 1). Upon relating with Zn deficiency (<65 μg/dL), majority of the patients (n=42, 84%) had neuropathy, while among diabetic patients with normal Zn level (>65 μg/dL), only 61.9% (n=26) patients had neuropathy. A statistically significant association was observed between zinc levels and diabetic neuropathy (p <0.001) (Table 2). In line with our results, a study conducted by Hussain et al. [15] further emphasizing the diverse nature of the current study conducted across different nations. The current investigation reveals patients with Zn deficiency (<65 μg/dL) had significantly higher rate of diabetic retinopathy (n=36, 72%) as compared to the patients with no Zn deficiency (>65 μg/dL) (n=17, 40.5%), also a higher proportion of patients with Zn deficiency (<65 μg/dL) had diabetic nephropathy (n=33, 66%) as compared to patients with no Zn deficiency (>65 μg/dL) (Table 2). Therefore, Type 2 DM patients with Zn deficiency had higher prevalence of microvascular complications as compared with diabetic patients with no zinc deficiency drawing a parallel with the study conducted by Luo et al. [16], further corroborating the reliability of the results achieved in our study. Mechanistically, as the human body cannot store Zn, it has to depend on daily dietary zinc intake to maintain homeostasis. As insulin maintains glucose homeostasis by phosphoinositide 3’-kinase (PI3K)/Akt pathway, Zn ions activate this cascade, resulting in glucose uptake and glycogen synthesis, thus decreasing blood glucose. Consequently, Zn deficiency increases blood glucose, resulting in all its associated complications chronically. 

Strengths and limitations

To our knowledge, this is the first study to examine the prevalence of Zn deficiency (<65 μg/dL) in Type 2 DM with glycemic control per se and assess its association with microvascular complications such as diabetic neuropathy, retinopathy, and nephropathy. In accordance with our selection criteria, we included only clinically diagnosed Type 2 DM excluding Type 1 DM and prediabetes; we admit that this might not fully account for some variability in our results. The generalizability of our findings is limited as we extracted data only from patients attending our hospital, so our representative data were exclusively from convenient sampling. The Zn estimation is limited to the demographics of Uttarakhand residents' dietary habits. Hence, the observed association between dietary Zn is specific to the region and thus cannot be generalized to other states of India and other populations. We tried to adjust for all potential confounders; however, residual confounding may have biased our results. 

## Conclusions

The study highlighted the high prevalence of Zn deficiency among Type 2 DM patients and established significant correlations between low Zn levels (<65 μg/dL) and poor glycemic control, as well as higher incidences of neuropathy, retinopathy, and nephropathy. These findings suggest that Zn status should be considered in the management and treatment strategies for Type 2 DM patients to potentially mitigate complications and improve outcomes. It is imperative that clinicians understand the optimal dosage of Zn for anti-hyperglycemic effects and potential side effects but also the interactions of Zn with other Type 2 DM complications, which warrants further investigation. 
